# High-degree atrioventricular block in acute ethanol poisoning: a case report

**DOI:** 10.4076/1757-1626-2-8559

**Published:** 2009-09-09

**Authors:** Miran Brvar, Matjaz Bunc

**Affiliations:** 1 Poison Control Centre, University Medical Centre LjubljanaZaloška cesta 7, 1000 LjubljanaSlovenia; 2 Department for Cardiology, University Medical Centre LjubljanaZaloška cesta 7, 1000 LjubljanaSlovenia

## Abstract

**Introduction:**

Acute ethanol ingestion can prolong the PR interval, but searching Medline, we have found only one report of Wenckebach-type atrioventricular block in ethanol poisoning. We present a high-degree atrioventricular block in an ethanol-poisoned patient.

**Case presentation:**

A 17-year-old woman with a non-contributory medical history ingested 3dcl of vodka and was found comatose. On arrival she was somnolent with nausea, tympanic temperature 36.0°C, pulse 70 counts/min, blood pressure 90/60 mmHg, respiratory rate 12 counts/min and SpO_2_ 96% on room air. Her blood ethanol level was 130 mg/dL; other blood laboratory test results were normal. ECG revealed sinus rhythm, first-degree atrioventricular block with a PR interval of 0.32 seconds and intermittent second- and third-degree atrioventricular blocks with up to 4-second-long pauses that appeared 15-30 seconds after each vomiting. She was given thiethylperazine and vomiting resolved within an hour. ECG 12 hours after admission revealed a first-degree atrioventricular block with a PR interval of 0.24 seconds. One month later Holter monitor showed a sinus rhythm and first-degree atrioventricular block with a PR interval of 0.21 seconds. Vagal maneuvers did not provoke high-degree atrioventricular block. The echocardiogram was normal.

**Conclusion:**

Acute ethanol poisoning has the potential to prolong the PR interval in adults with first-degree atrioventricular block and provoke intermittent second- and third-degree atrioventricular blocks, possibly by its direct inhibitory action on the conduction system and increasing parasympathetic tone due to nausea and vomiting.

## Introduction

Ethanol abuse has been associated with tachyarrhythmias and increased mortality [1,2]. In young healthy volunteers acute ethanol ingestion increases heart rate [3,4], and prolongs the QTc interval due to prolongation of ventricular repolarisation [5-7], with a consequent increased risk of life-threatening re-entrant ventricular tachyarrhythmia and sudden cardiac death [6]. The mechanism of QTc prolongation in acute ethanol poisoning is still not completely understood, but it is probably primarily the result of ethanol direct inhibitory action on the cardiac conduction system and myocardium, since it cannot be explained only by changes in autonomic nervous activity, myocardial ischemia and electrolyte abnormalities [7,8].

Interestingly, acute ethanol poisoning in otherwise healthy adults has also been associated with bradycardia. In studies with healthy volunteers it was shown that ethanol ingestion cause prolongation of the PR interval in electrocardiogram (ECG) [5,6], and in chronic alcoholics complete atrioventricular block in ECG, prolongation of His-ventricular conduction in electrophysiological studies and fibrofatty infiltration of the conduction system on autopsies were observed [1,8,9]. However, searching Medline, we have found only one report of bradycardia due to Wenckebach-type atrioventricular block in severely ethanol poisoned young, healthy adult [10].

We present a patient with third-degree atrioventricular block in moderate acute ethanol poisoning.

## Case presentation

**Figure 1.**
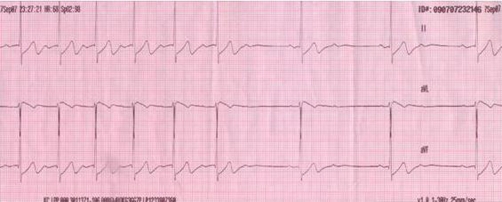
An ECG record showing sinus rhythm, first-degree atrioventricular block with PR interval of 0.32 seconds and intermittent second-degree atrioventricular block 15-30 seconds after vomiting in 17-year-old ethanol poisoned girl.

**Figure 2.**
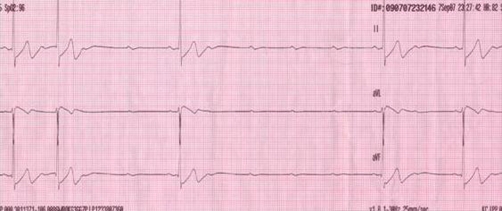
An ECG record showing intermittent third-degree atrioventricular block with up to 4 second-long pauses 15-30 seconds after vomiting in 17-year-old ethanol poisoned girl.

A 17-year-old woman of Slovene ethnic origin with a non-contributory medical history ingested 3dcl of vodka and was found comatose. On arrival at the Emergency Department she was somnolent with tympanic temperature 36.0°C, pulse 70 counts/min, blood pressure 90/60 mmHg, respiratory rate 12 counts/min and SpO_2_ 96% on room air. She had nausea and vomited several times. Her blood ethanol level was 130 mg/dL; other blood laboratory test results, such as glucose, electrolytes, myoglobine, troponine I and liver tests, were normal. ECG revealed sinus rhythm, first-degree atrioventricular block with a PR interval of 0.32 seconds and intermittent second- and third-degree atrioventricular blocks with up to 4-second-long pauses that appeared 15-30 seconds after each vomiting (Figures 1 and 2). She was given oxygen, thiethylperazine, pantoprazolum and a continuous infusion of glucose. Nausea and vomiting resolved within an hour and during subsequent treatment she had a normal heart rate. On discharge 12 hours after admission, ECG revealed a sinus rhythm and first-degree atrioventricular block with a PR interval of 0.24 seconds. Subsequent toxicology analysis by gas chromatography coupled to mass spectrometry revealed no drugs in the patient’s blood and urine samples. One month later she was re-examined and she denied any symptom of bradycardia. A sinus rhythm and first-degree atrioventricular block with a PR interval of 0.21 seconds was noted on ECG. Holter monitor revealed a sinus rhythm with a frequency of 50-150/min and first-degree atrioventricular block with a PR interval of 0.21 seconds. Vagal maneuvers did not provoke second- or third-degree atrioventricular block. The echocardiogram was normal. The serology for borealis and antinuclear antibodies was negative.

## Discussion

Acute ethanol poisoning has the potential to prolong the PR interval in young healthy adults with first-degree atrioventricular block and provoke intermittent second- and third-degree atrioventricular blocks most probably by its direct inhibitory action on the conduction system and increased vagally mediated parasympathetic tone due to nausea and vomiting. Direct inhibitory action of ethanol on the cardiac conduction system was shown in animal [11], and human studies [12], and a vagally mediated conduction disturbance was observed during electrophysiological studies [8,13]. Furthermore, an initial decrease in sympathetic nerve activity was observed in volunteers after moderate ethanol ingestion, that could also contribute to bradycardia [3,4].

Although ethanol abuse is very common, bradycardia and atrioventricular block in acute ethanol poisoning is rarely described in medical literature. The reason might be in the absence or overlapping of atrioventricular block symptoms with other symptoms commonly presented in ethanol poisoning, such as dizziness, falling down or loss of consciousness. Furthermore, atrioventricular conduction delay after ethanol ingestion might become clinically obvious only in persons with preexisting prolonged cardiac conduction reflecting in first-degree atrioventricular block, which is reported in 1% of a young, healthy population. In addition, the arrhythmogenic effects of ethanol could also be intensified in persons with hidden cardiac lesions and chronic alcoholics with degenerative changes in the conduction system [1,9].

Accordingly it would be interesting to study ECG changes and associated symptoms in ethanol poisoned patients with preexisting conduction abnormalities, because dizziness or loss of consciousness in these patients might also be due to intermittent bradycardia. In adults with known conduction abnormalities cautious ethanol drinking should be recommended.

## Conclusion

Acute ethanol poisoning has the potential to prolong the PR interval in young, healthy adults with preexisting first-degree atrioventricular block and provoke intermittent second- and third-degree atrioventricular blocks.
